# Lotus Root Polysaccharide-Phenol Complexes: Interaction, Structure, Antioxidant, and Anti-Inflammatory Activities

**DOI:** 10.3390/foods12030577

**Published:** 2023-01-28

**Authors:** Kaidi Peng, Yin Li, Ying Sun, Wei Xu, Hongxun Wang, Rui Zhang, Yang Yi

**Affiliations:** 1 Hubei Key Laboratory for Processing and Transformation of Agricultural Products, College of Food Science and Engineering, Wuhan Polytechnic University, Wuhan 430023, China; 2 Hubei Industrial Technology Research Institute of Jingchu Special Foods, Jingzhou 434000, China; 3 National R & D Center for Serich Agricultural Products Processing, School of Modern Industry for Selenium Science and Engineering, Wuhan Polytechnic University, Wuhan 430023, China

**Keywords:** lotus root polysaccharides, gallic acid, epigallocatechin, interaction, antioxidant, anti-inflammatory

## Abstract

This research aimed to explore the interaction between lotus root polysaccharides (LRPs) and phenolic compounds, and to study the effects of phenolic binding on the structural and functional properties of LRPs. The influences of pH, temperature, and NaCl and phenol concentration on the binding ratio of gallic acid (GA)/epigallocatechin (EGC) to LRPs were evaluated. LRP-GA/EGC complexes with different phenolic binding amounts were then prepared and characterized via ultraviolet–visible (UV–Vis) and Fourier-transform infrared (FTIR) spectroscopy, and average molecular weight (MW) measurements. The results suggest that hydrogen bonds contributed to the binding of GA/EGC and LRPs. The phenolic binding led to significant changes in the structure and MW of LRPs. Moreover, antioxidant activity and the macrophage-stimulating effect of LRPs were improved after binding with GA/EGC, depending on the binding amount and type of polyphenol. Interestingly, LRP-GA/EGC complexes with polyphenol binding amounts of 105.4 mg/g and 50.71 mg/g, respectively, showed better stimulation effects on the anti-inflammatory cytokine IL10 secretion of macrophages when compared to LRPs. These results show the great potential of phenolic binding to be applied to improve the structure and functional activity of LRPs.

## 1. Introduction

Lotus (*Nelumbo nucifera* Gaertn.) root is one of the most popular aquatic vegetables cultivated and consumed in Southeast Asia. It has attracted considerable attention due to its edible properties and therapeutic potential [1]. Polysaccharides are suggested to be important functional factors that contribute to its potential health benefits, e.g., antioxidant, immunomodulatory, and anti-obesity activities [2,3]. Lotus root also contains phenolic compounds that are related to the benefits. Recently, interesting results have been reported that polysaccharides could interact with polyphenols spontaneously when they are physically mixed, leading to the improved bioaccessibility, stability, and flavor of end products [4,5]. For example, Tudorache and Bordenave [6] observed that phenolic compounds mediated the colloidal aggregation and decreased the pseudo-first behavior of polysaccharide solutions. Jakobek and Matić [7] reported that associating with dietary fiber might control the amount of bioaccessible polyphenols in the upper or lower parts of the digestive tract. Concerning lotus root polysaccharides (LRPs), these have a great opportunity to contact and interact with polyphenols whenever they are released from the cell during lotus root harvesting, processing, and eating.

Polysaccharides may interact with polyphenols by covalent and non-covalent interactions [8]. Non-covalent interactions occur more easily during common processing conditions and have garnered more interest from researchers [9]. It was proposed that non-covalent interactions are mediated by hydrogen bonds, hydrophobic interactions, and electrostatic interactions. The intensity of non-covalent interactions is likely to be dependent on the physical–chemical properties of substrates as well as environmental factors (pH, temperature, and ionic strength). Studies have reported that hydrogen bonds are the main driving force for the complex formation between epigallocatechin gallate and oat β-glucan [10], whereas both hydrogen bonding and electrostatic forces are responsible for the interactions between ferulic acid and arabinan-rich pectic polysaccharides from rapeseed meal [9].

However, the nature of the binding mechanism between LRPs and polyphenols, as well as the positive effects on the nutritional value of LRPs, have rarely been explored. In this study, we aimed to reveal the interaction mechanism of LRPs and polyphenols, study the effects of phenolic binding on the structure, antioxidant, and macrophage-stimulating activities of LRPs. Polysaccharides were extracted from lotus root and GA/EGC were selected as the representatives of phenolic compounds. The effects of environment factors (pH, temperature, and NaCl concentration) on the interactions between LRPs and GA/EGC were evaluated. In addition, the complexes of LRP-GA/EGC were prepared with different binding ratios of GA/EGC and instrumentally characterized, and the effects of binding GA/EGC on the antioxidant and anti-immunomodulatory effects of LRPs were examined.

## 2. Materials and Methods

### 2.1. Materials and Chemicals

**Figure 1:**
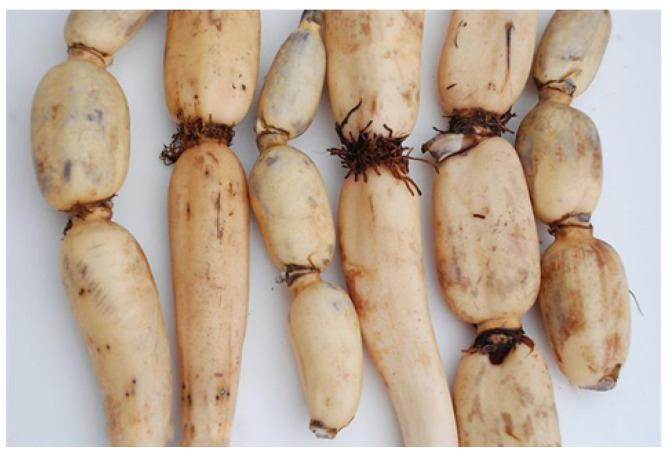
Image of lotus roots.

Fresh lotus roots (cultivar Elian No. 5) used in this work were purchased from Wuhan Jinshui-qiliang Agricultural Products Co., Ltd. (Wuhan, China). Lotus roots (Figure 1) were washed with tap-water to remove mud, and knots were discarded. Roots were then peeled, sliced, and dried in an electric thermostatic drying oven (DHG-9140A, Shanghai Yiheng Scientific Instrument Co., Ltd., Shanghai, China) at 65 °C for 12 h. The dried slices were ground into fine powder and stored in a desiccator at room temperature for further use.

Gallic acid (≥99%) and epigallocatechin (≥98%) were purchased from Shanghai Macklin Biochemical Co., Ltd. (Shanghai, China). Coomassie brilliant blue assay kit and ferric reducing antioxidant power (FRAP) kit were obtained from Shanghai Beyotime Technology (Shanghai, China). Gibco Dulbecco’s Modified Eagle Medium (DMEM) and phosphate-buffered saline (PBS) were purchased from Hyclone Company (Logan, American). Lipopolysaccharides were obtained from Beijing Biotopped Co., Ltd. (Beijing, China). RNA extraction solution, primers, and first strand cNDA synthesis kit were purchased from Wuhan Servicebio Technology Co., Ltd. (Wuhan, China). Mouse RAW264.7 macrophages were provided by Biosea Biotechnology Co. Ltd. (Wuhan, China). Folin–Ciocalteu reagent, 2,2-diphenyl-1-picrylhydrazyl (DPPH), and other analytic reagents were purchased from Sinopharm Chemical Reagent Co., Ltd. (Shanghai, China).

### 2.2. Isolation and Proximate Composition of Lotus Root Polysaccharides

Lotus root polysaccharides (LRPs) were prepared according to a previous study with minor modifications [11]. Briefly, dried lotus root powder was extracted with distilled water (solid:liquid ratio of 1:8, *w*/*v*) in a water bath with ultrasound treatment at 200 W for 5 min and then placed in a 90 °C water bath with stirring for 3 h. The mixture was centrifuged at 4500 rpm for 10 min. The supernatant was mixed with 30% (*v*/*v*) ethanol distilled water solution, and incubated at 4 °C for 3 h to precipitate starches. The non-starch solution was obtained by centrifugation at 4500 rpm for 6 min to remove the sediment and confirmed using iodide/iodine reagent. Then, the non-starch solution was concentrated using a rotary evaporator under vacuum at 55 °C and subjected to the Sevage method for the removal of proteins. The concentrated solution was precipitated with three-fold volumes of 95% (*v*/*v*) ethanol distilled water solution at 4 °C for 12 h. The precipitates were collected by centrifugation at 4500 rpm for 6 min, re-dissolved in distilled water, concentrated again as mentioned above, and lyophilized to obtain LRPs.

The total sugar content was measured according to the phenol sulfuric acid method [12] and presented as glucose equivalents [13]. The reducing sugar content was determined by the 3,5-dinitrosalicylic acid (DNS) method [14]. The protein content was evaluated using a Coomassie Brilliant Blue staining kit according to the manufacturer’s instructions. The total phenolic content was determined by the Folin–Ciocalteu method [15]. Briefly, 0.125 mL sample solution, 0.625 mL distilled water, and 0.125 mL Folin–Ciocalteu reagent were mixed well and incubated in the dark at room temperature for 10 min. Then, 1.25 mL 7% NaCO${}_{3}$ was added, and the mixture was kept in the dark for 90 min, followed by absorbance measurement using a spectrometer (Shimadzu, UV-1800, Kyoto, Japan) at 760 nm. A calibration curve was established using GA (20–100 μg/mL ) as the standard. Each sample measurement was performed in three replicates.

### 2.3. Preparation of LRP-GA and LRP-EGC Complexes

Polysaccharide-phenol complexes were prepared following the method of Li et al. [16] with slight modifications. LRP (8 mL, 1 mg/mL), GA, and EGC solutions (4 mL, varying concentrations) were prepared. LRP solutions were made at low concentration to allow complete dissolving in distilled water and avoid self-aggregation or phenolic-induced aggregation [17]. The LRP & GA and LRP & EGC mixtures were obtained by mixing corresponding solutions in a 30 mL screw-capped glass bottle, and then stirring for 30 min at different pH, temperatures, or NaCl concentrations. Mixture solutions of 10 mL were transferred to pre-treated dialysis bags (MWCO1000, Spectrum Laboratories, Compton, CA, USA), followed by immersing in 190 mL distilled water (equal to 20-fold dilution of mixture solution) and incubating for 4 h at the same temperature as before. The reserved solution in dialysis bags was collected and freeze-dried as polysaccharide-phenol complexes. The varied preparation conditions were concentration of GA/EGC (0.5, 1, 2, 4, and 8 mg/mL), pH (3, 4, 5, 6, and 7, adjusted by 0.1 M HCl or 0.1 M NaOH), temperature (0, 15, 30, 45, and 60 °C) and NaCl concentration (0, 20, 40, 60, and 80 mmol/L).

After the dialysis bag was removed, the phenol concentration in the dialysate was determined by the Folin–Ciocalteu method. The binding ratio (Y), in terms of the amount of GA/EGC (mg) bound per gram of LRPs, was calculated by the following equation:

(1) $$Y=\left(M_{0}-CV\right)/M_{LRPS}$$

where *M*${}_{0}$ is the mass of the GA/EGC applied (mg), *C* is the phenol concentration in the dialysate (mg/mL), *V* is the total volume of the dialysis system (200 mL), and *M*${}_{LRPs}$ is the mass of LRPs applied (8 mg).

### 2.4. Characterization of LRP-GA and LRP-EGC Complexes

The LRP-GA (LRP-GA${}_{1}$, LRP-GA${}_{2}$, LRP-GA${}_{3}$) and LRP-EGC (LRP-EGC${}_{1}$, LRP-EGC${}_{2}$, LRP-EGC${}_{3}$) complexes with different binding ratios of GA/EGC to LRPs were prepared at optimum conditions and named as shown in Table 1.

#### 2.4.1. Ultraviolet–Visible (UV–Vis) Spectrum Analysis

The UV–Vis spectra of fifteen samples (LRPs, GA, EGC, complexes, and their physical mixtures) were determined at a wavelength range of 190–500 nm using a UV–Vis spectrophotometer (A360, Aoyi Instruments Shanghai Co., Ltd., Shanghai, China). The aqueous solutions of LRPs and phenolic compounds were prepared with distilled water at the concentration of 0.02 mg/mL. The LRP-GA${}_{1}$, LRP-GA${}_{2}$, and LRP-GA${}_{3}$ were dissolved in distilled water at 0.02 mg/mL, 0.05 mg/mL, and 0.1 mg/L, respectively (same preparation concentrations for LRP-EGC complexes). The physical mixtures of LRP & GA and LRP & EGC were prepared by mixing the LRPs and phenol solution with the same molar ratio as the complexes. All the samples were prepared and then scanned immediately.

#### 2.4.2. Fourier-Transform Infrared (FTIR) Spectrum Analysis

The FTIR spectra of samples were measured using an FTIR spectrophotometer (Nexus 5DXC FT-IR, Thermo Nicolet, Madison, USA). The samples (2 mg) were ground with KBr and pressed into pellets for FTIR measurement in the frequency range 4000–400 cm${}^{-1}$, with a scanning number of 32 and a resolution of 4 cm${}^{-1}$ [18].

#### 2.4.3. High-Performance Size-Exclusion Chromatography

The molecular weight (MW) distributions of LRPs and LRP-phenol complexes were evaluated by the method of Yi et al. [11] and using a high-performance size-exclusion chromatograph (HPSEC) equipped with a column of Aglient PL aquagel OH. Samples were dissolved in the mobile phase with a concentration of 0.2 mol/L, and then centrifuged at 13,000 rpm for 5 min. The supernatant was filtered through 0.22 μm film and used for injection. It was eluted by the mobile phase of 0.2 mol/L CH${}_{3}$COOHNH${}_{4}$ solution at a flow rate of 0.7 mL/min and column temperature of 30 °C. The peaks were determined using a multi-angle laser-light scattering detector (DAWN HELEOS-II 18, Wyatt Technology Co., Santa Barbara, CA, USA) and refractive index detector (Optilab rEX, Wyatt Technology Co.).

### 2.5. Antioxidant Activities of LRP-GA and LRP-EGC Complexes

The 2,2-diphenyl-1-picrylhydrazyl (DPPH) radical scavenging ability was assessed based on the method reported by Rodríguez et al. [19]. In brief, 100 μL aqueous solution of sample (varying concentrations ranging from 0.1 mg/mL to 1.0 mg/mL), 1 mL DPPH (100 μmol/L) solution, and 1 mL distilled water were mixed well and then incubated for 30 min in the dark. After reaction, the absorbance of the mixture was determined at 517 nm. Similarly, a control was prepared with methanol instead of sample. Blank sample was obtained using methanol instead of DPPH solution. The DPPH radical scavenging ability was expressed as the scavenging ratio (S, %) and calculated by the equation:

(2) $$S\;=\;[1-\left(A_{s}-A_{b}\right)/A_{c}]\times 100$$

where A${}_{c}$, A${}_{s}$, and A${}_{b}$ are the absorbance of the control, sample, and blank sample, respectively. The sample concentration required for a 50% (IC${}_{50}$, mg/mL) scavenging ratio against DPPH radical was calculated according to the linear equation of concentration vs. scavenging rate.

The evaluation of ferric reducing antioxidant power (FRAP) was performed using the FRAP kit according to its instruction. The results were expressed as millimoles of Fe${}^{2+}$ equivalents per 1 g of sample.

### 2.6. Immunomodulatory Activity of LRP-GA and LRP-EGC Complexes

The immunomodulatory activity was measured by the NO production and the mRNA expression levels of tumor necrosis factor-α (TNF-α) and interleukin-10 (IL-10). In detail, RAW264.7 macrophage suspension was prepared with DMEM medium at a density of 5 × 10${}^{5}$ cells/mL and then placed in 48-well culture plates (500 μL/well). Cells were incubated at 37 °C in a 5% CO${}_{2}$ incubator for 3 h, followed by removing the supernatant. Adherent cells were obtained and re-incubated in medium alone (control group) or medium containing samples and/or LPS. All aqueous solutions of samples and LPS were prepared with DMEM medium. Aqueous solutions of LRPs and LRP-phenol complexes were prepared with the same LRP concentration of 200 μg/mL; aqueous solution of LPS was prepared with a concentration of 500 ng/mL and used as a positive control. After incubation for 24 h and 48 h, the NO concentration in the macrophage culture supernatant was determined by the Greiss method [12,13].

Cells incubated for 24 h with the same stimulation procedures mentioned above were collected, washed with phosphate buffered saline (PBS) twice, and used for extraction of total cellular RNA. Total cellular RNA was isolated using the RNA extraction solution (Servicebio, Wuhan, China) and treated following the manufacturer’s protocol. cDNA was synthesized with 2 μg total cellular RNA using hexamers for priming and a first-strand cNDA synthesis kit (Servicebio, Wuhan, China) according to the manufacturer’s instructions. Quantitative real-time PCR was performed using a Real-time PCR System (Thermo fisher scientific, USA) with 2 × SYBR Green qPCR Master Mix (None ROX, Servicebio, Wuhan, China) following the manufacturer’s recommendations. The primers used in the reaction and the glyceraldehyde-3-phosphate dehydrogenase (GAPDH) gene serving as endogenous references were as follows: 5’-CCTCGTCCCGTAGACAAAATG-3’ (sense primer) and 5’ TGA GGTCAATGAAGGGGTCGT-3’ (antisense primer) for GAPDH, 5’-CTC TTCTGTCTACTGAACTTCGGG -3’ (sense primer) and 5’-GGTGGTTTGTGAGTGTGAGGGT-3’ (antisense primer) for TNF-α, 5’-TGCCAAGCCTTATCGG AAATG-3’ (sense primer) and 5’-AAATCACTCTTCACCTGCTCCAC-3’ (antisense primer) for IL-10 [20].

### 2.7. Statistical Analysis

All the experiments were performed in triplicate, and the data were recorded as mean ± standard deviation. Statistical analysis was performed with SPSS 19.0 software (IBM, Armonk, NY, USA), and the significant differences (*p* < 0.05) between groups were estimated with one-way ANOVA followed by the Student–Newman–Keuls test.

## 3. Results and Discussion

### 3.1. Physicochemical Properties of LRPs

The physicochemical properties of LRP powders were measured after water extraction, 30% alcohol precipitation, deproteinization, 75% alcohol precipitation, and lyophilization. The total sugar content of LRPs was 87.48%, reducing sugar content was 2.79%, polyphenol content was 1.13%, and protein content was 0.20%.

### 3.2. Factors That Influence the Binding Ratio of GA/EGC to LRPs

The binding interactions between polyphenols and polysaccharides were mostly non-covalent under normal processing conditions [18], and were mainly mediated by hydrogen bonds, hydrophobic interactions, and ion interactions. These could be influenced by environmental conditions like compounds, pH, temperature, and ionic strength [16]. Thus, the individual effects of pH, temperature, concentration of NaCl, and concentration of GA/EGC on the binding ratio were investigated to determine the complexation mechanism between GA/EGC and LRPs.

#### 3.2.1. Effect of pH

**Figure 2:**
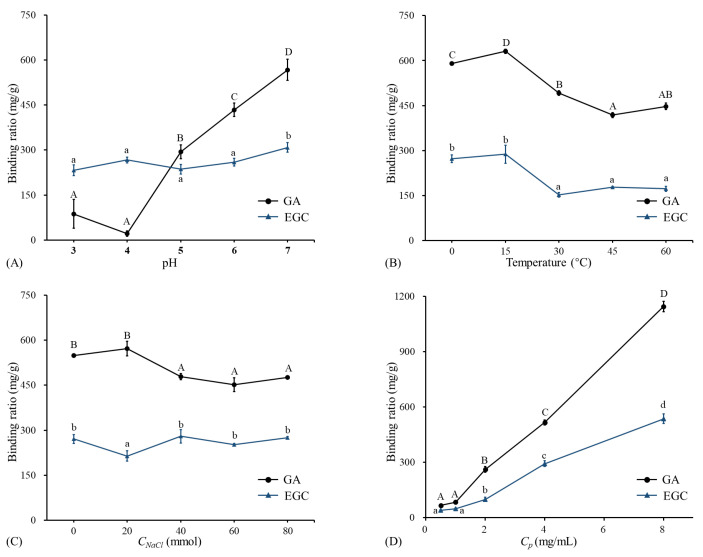
The binding ratios of GA and EGC to LRPs at different pH, temperatures, NaCl concentrations, and phenol concentrations. (**A**) The binding ratios of GA and EGC to LRPs at different pH, temperature of 15 °C, and mass ratio of GA/EGC to LRPs of 2:1; (**B**) the binding ratios of GA and EGC to LRPs at different temperatures, pH 7, and mass ratio of GA/EGC to LRPs of 2:1; (**C**) the binding ratios of GA and EGC to LRPs at different NaCl concentrations, pH 7, temperature of 15 °C, and mass ratio of GA/EGC to LRPs of 2:1; (**D**) the binding ratios of GA and EGC to LRPs at different C${}_{p}$ phenol concentrations (different mass ratios of GA/EGC to LRPs of 1:4, 1:2, 1:1, 2:1, 4:1), pH 7, and temperature of 15 °C. Means of the samples with different letters (A–D, a–d) differ significantly (*p* < 0.05).

The binding ratios of GA/EGC to LRPs at different pH (2–7) are shown in Figure 2A. The binding ratio of GA to LRPs slightly decreased from pH 3 to 4, and then significantly increased with pH increasing from 4 to 7. The highest binding ratio value of 567.25 ± 34.89 mg/g was observed at pH 7. Badhani and Kakkar [21] reported that gallic acid had a pKa of 5 and existed in the anionic form in the pH range 5 to 9 with the deprotonation of the carboxy-group or hydroxy-group [22].

According to previous studies, if polyphenols are absorbed onto polysaccharides via electrostatic interaction, the electrostatic repulsion increases with pH increasing and the polyphenols absorbed can be reduced [9,23]. However, results in this study were not shown in this way. This implies that electrostatic interaction may not play an important role in the binding of GA and LRPs. Phan et al. [23] also obtained similar results regarding the complexation of ferulic acid and cellulose. Studies reported that the higher the ratio of deprotonated/protonated carboxyl groups (COO${}^{-}$/COOH), the higher the repulsion between dietary fiber molecules, which stabilized those molecules as micelles and led to better entrapment of hydrophobic polyphenol curcumin [7,24]. For EGC, the binding ratio remained stable from pH 3 to 6, and showed a significant increase from pH 6 to 7. EGC (pKa is 7.87) was protonated in the pH range 2–7 [25]. With increasing pH, the decreased H${}^{+}$ had little effect on the binding, and then due to the increased neutral fraction, the binding amount had the highest value (308.27 ± 15.96 mg/g) at pH 7. This indicated that electrostatic interactions made little sense in the association between EGC and LRPs. Similarly, Le Bourvellec et al. [26] reported that the binding of procyanidins and apple cell-wall material had no significant variation within the pH range 2–7. The pH had a different effect on the binding of GA and EGC to LRPs. This is consistent with previous literature reporting that the pH effect on the combination of polysaccharides and polyphenols is greatly dependent on the type of polyphenol [27].

#### 3.2.2. Effect of Temperature

The binding ratios of GA/EGC to LRPs at different temperatures are shown in Figure 2B. The binding amount of GA significantly increased with temperature increasing from 0 °C to 15 °C, and then significantly decreased with temperature increasing from 15 °C to 45 °C, and showed a slight increase from 45 °C to 60 °C. The binding amounts of EGC to LRPs had no change with temperature increasing from 0 °C to 15 °C, and then sharply decreased when the temperature increased from 15 °C to 30 °C, and remained stable with the temperature continually increasing from 30 °C to 60 °C. Both GA and EGC had larger binding ratios (630.96 ± 6.77 mg/g and 288.13 ± 30.13 mg/g, respectively) to LRPs at 15 °C than at other temperatures. It was inferred that the binding of GA/EGC to LRPs was not an entire endothermic process or physical adsorption process [28]. When the temperature rose to 15 °C, the notable decrease of the binding amount with increasing temperature implied the hydrogen bonding would drive the sorption of GA/EGC to LRPs [9]. A similar observation was reported by Wu et al. [28] that increasing temperature from 20 °C to 30 °C increased the adsorption of oat to β-glucan, while the increase in temperature from 30 °C to 60 °C adversely affected the binding.

#### 3.2.3. Effect of NaCl Concentration

The data in Figure 2C show the binding ratio of GA and EGC to LRPs at different NaCl concentrations. In general, by increasing the NaCl concentration, the binding ratio of GA to LRPs first increased slightly (*p* > 0.05), reached its maximum point (572.31 ± 24.77 mg/g) at an NaCl concentration of 20 mmol, and then kept stable. Conversely, the binding ratio of EGC at an NaCl concentration of 20 mmol appeared to have a lower binding amount than at lower or higher NaCl concentration values. Theoretically, if hydrophobic interactions are formed between the hydrophobic regions of LRPs and the hydrophobic groups (e.g., aromatic rings) of GA/EGC, one of their characteristics is that their strength increases with an increase in ionic strength [23,29]. However, this phenomenon was not observed in this study. This attested that the hydrophobic interactions between GA/EGC and LRPs were weak. Previous research showed similar results that hydrophobic interactions were not a major contributor between ferulic acid and arabinan-rich pectic polysaccharide from rapeseed meal [9].

#### 3.2.4. Effect of Phenol Concentration

As shown in Figure 2D, the binding amount of GA/EGC to LRPs increased approximately linearly with increasing the initial GA/EGC concentration from 0.5 mg/mL to 8 mg/mL. This suggests there was equilibrium between free and bound phenol, whereas the increase in concentration could result in the increase in binding capacity [8]. Because of the limited solubility of GA/EGC in distilled water, the experiment could not be conducted at higher phenol concentrations. GA/EGC had maximum bound ratio values of 1144.18 ± 27.50 and 535.98 ± 26.16 mg/g, respectively. At the same preparation conditions, GA showed better binding capacity to LRPs compared to EGC. Both GA and EGC had hydroxyl groups as well as aromatic rings, whereas the differing molecular weights, degrees of galloylation, and number and position of various functional groups could lead to their varied adsorption amounts to LRPs.

### 3.3. Characterization of LRP-GA and LRP-EGC Complexes

The interactions between LRPs and phenolic compounds (GA and EGC) as well as the structural properties of LRP-GA and LRP-EGC complexes with different binding amounts of GA/EGC were evaluated. LRP-GA${}_{1}$, LRP-GA${}_{2}$, LRP-GA${}_{3}$, LRP-EGC${}_{1}$, LRP-EGC${}_{2}$, and LRP-EGC${}_{3}$ complexes were prepared, respectively, with binding ratios of 1115.05, 336.14, 105.42, 698.71, 169.09, and 50.71 mg/g. The corresponding mass ratios of bound GA and EGC to LRP-GA/EGC complexes were 52.72%, 25.16%, 9.62%, 41.13%, 14.46%, and 4.83%, respectively.

#### 3.3.1. UV–Vis Spectrum Analysis

**Figure 3:**
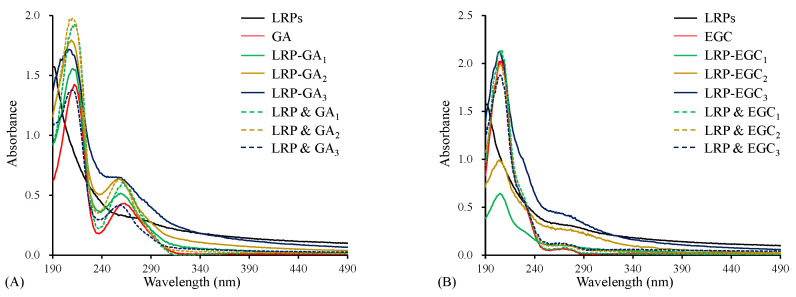
The UV–Vis spectra of LRPs, polyphenols, LRP-phenol complexes, and their physical mixtures. LRPs, GA, LRP-GA complexes, and their physical mixture (**A**); LRPs, EGC, LRP-EGC complexes, and their physical mixture (**B**).

The UV–Vis spectrum of LRPs had the characteristic absorption of unsaturated carbonyl and carboxyl groups in the wavelength range 190–220 nm, and weak absorption of a few proteins incorporated in LRPs at 280 nm (Figure 3A) [13]. Both GA and LRP & GA mixtures had strong absorption at around 210 nm and 260 nm (Figure 3A), due to the bathochromic effect of hydroxyl groups on the aromatic ring [30]. For LRP-GA complexes, all showed the similar strong absorption peak at 210 nm, but a weaker peak at 260 nm compared to their corresponding physical mixtures. Particularly, the peak at 260 nm was not obvious for the LRP-GA${}_{3}$ complex. Similarly, the absorption peak was observed at 270 nm on the UV–Vis spectrum of EGC and LRP & EGC mixtures (Figure 3B), and it disappeared in the LRP-EGC complexes. It was implied that the hydroxyl group of GA/EGC was involved in the interaction with LRPs. Similarly, Li et al. [16] found that the chromatographic peaks of GA/catechin disappeared in the polysaccharide-GA/catechin complexes.

#### 3.3.2. FTIR Spectrum Analysis

**Figure 4:**
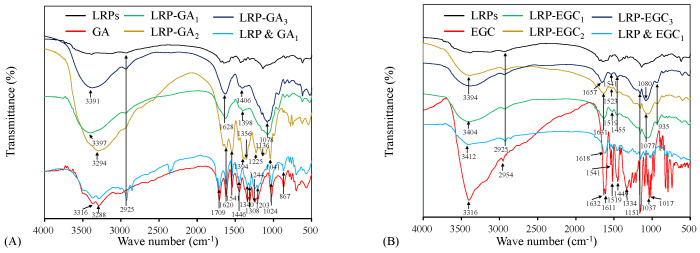
FTIR spectra of LRPs, polyphenols, LRP-phenol complexes, and their physical mixtures. LRPs, GA, LRP-GA complexes, and their physical mixture (**A**); LRPs, EGC, LRP-EGC complexes, and their physical mixture (**B**).

FTIR spectrum analysis was carried out to characterize the binding of GA and EGC on LRPs. Figure 4A shows the FTIR spectrum of LRPs, GA, their physical mixture, LRP-GA${}_{1}$, LRP-GA${}_{2}$, and LRP-GA${}_{3}$ complexes. The GA spectrum exhibited typical broad absorption bands of O-H stretching vibrations (3316 cm${}^{-1}$ and 3288 cm${}^{-1}$), C=O stretching vibration (1709 cm${}^{-1}$), aromatic ring with substitutions (1620 cm${}^{-1}$, 1541 cm${}^{-1}$), C-C and C-O stretching vibrations (1446 cm${}^{-1}$, 1200–1300 cm${}^{-1}$, 1024 cm${}^{-1}$), and C-H bending vibration (867 cm${}^{-1}$) [21,30,31]. The LRP & GA physical mixture had a similar FTIR spectrum to GA, except for the absorption peak at 2925 cm${}^{-1}$. The broad peak at 2925 cm${}^{-1}$ was attributed to the C-H stretching vibration of LRPs, which was also observed on the FTIR spectra of LRPs, LRP-GA${}_{1}$, LRP-GA${}_{2}$, and LRP-GA${}_{3}$ [11]. The O-H stretching vibration bands in LRP-GA complex spectrum (at 3397 cm${}^{-1}$, 3294 cm${}^{-1}$, and 3391 cm${}^{-1}$ for LRP-GA${}_{1}$, LRP-GA${}_{2}$, and LRP-GA${}_{3}$, respectively) were broader than that in the GA and LRP spectrum. It was reported that the O-H stretching band is a sensitive indicator of the strength of the hydrogen bond, and broadening of the O-H stretching band is often observed in strong hydrogen bonds [32]. Here, it was suggested that the hydrogen bond was formed between LRPs and GA. Compared to GA, the bands located at 1709 cm${}^{-1}$, 1541 cm${}^{-1}$, 1446 cm${}^{-1}$, 1244 cm${}^{-1}$, and 1203 cm${}^{-1}$ disappeared for LRP-GA complexes; the absorption peaks at 1340 cm${}^{-1}$ and 1308 cm${}^{-1}$ were attenuated and shifted, leading to absorption peaks at 1398 cm${}^{-1}$ and 1406 cm${}^{-1}$ on the spectra of LRP-GA${}_{2}$ and LRP-GA${}_{3}$, respectively. LRP-GA${}_{1}$, LRP-GA${}_{2}$, and LRP-GA${}_{3}$ spectra had characteristic peaks at 1041 cm${}^{-1}$, 1078 cm${}^{-1}$, and 1078 cm${}^{-1}$, respectively, which was likely related to the shift of the GA band at 1024 cm${}^{-1}$. The absorption intensity of bands at 897 cm${}^{-1}$ decreased.

The FTIR spectrum of EGC, physical mixture of LRPs and EGC, and LRP-EGC complexes are shown in Figure 4B. For EGC, the prominent peaks at 3316 cm${}^{-1}$ and 2954 cm${}^{-1}$ represent the stretching vibrations of O-H and C-H, respectively. The bands within 1400–1632 cm${}^{-1}$ and 1200–1334 cm${}^{-1}$ are related to aromatic rings [31,33,34]. Peaks observed within 1017–1151 cm${}^{-1}$ correspond to C-O stretching vibrations [35,36]. After binding to LRPs, the peak at 3316 cm${}^{-1}$ (O-H vibrational frequency) was shifted to a higher frequency, which was mainly due to the migration of the electron cloud in oxygen atoms on the phenol to a higher wavenumber [37]. The absorption peaks at 2954 cm${}^{-1}$ (C-H stretching vibrations) and 1200–1334 cm${}^{-1}$ (aromatic ring) disappeared. The intensity of the broad peaks at 1632, 1611, 1519, 1449, 1151, 1037, and 1017 cm${}^{-1}$ were shifted and diminished. The results imply that the interaction of LRPs and GA/EGC exists, which obviously altered the FTIR spectrum properties of LRPs and GA/EGC [16].

### 3.4. Molecular Weight (MW) Distributions of LRP-Phenol Complexes

**Figure 5:**
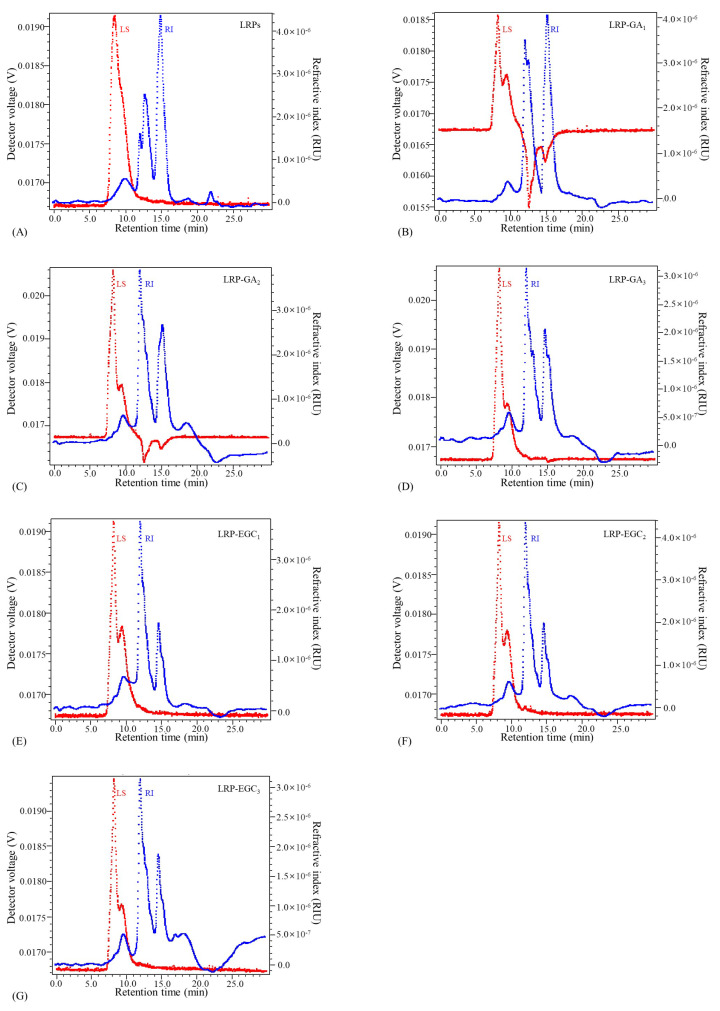
Elution profiles of LRPs and LRP-phenol complexes obtained by the HPSEC-MALL-RI method. LRPs(A);LRP-GA${}_{1}$(B); LRP-GA${}_{2}$(C); LRP-GA${}_{3}$(D); LRP-EGC${}_{1}$(E); LRP-EGC${}_{2}$(F); LRP-EGC${}_{3}$(G). Dotted line in blue presents the refractive index (RI) intensity, dotted line in red presents the light scattering (LS) intensity.

**Figure 6:**
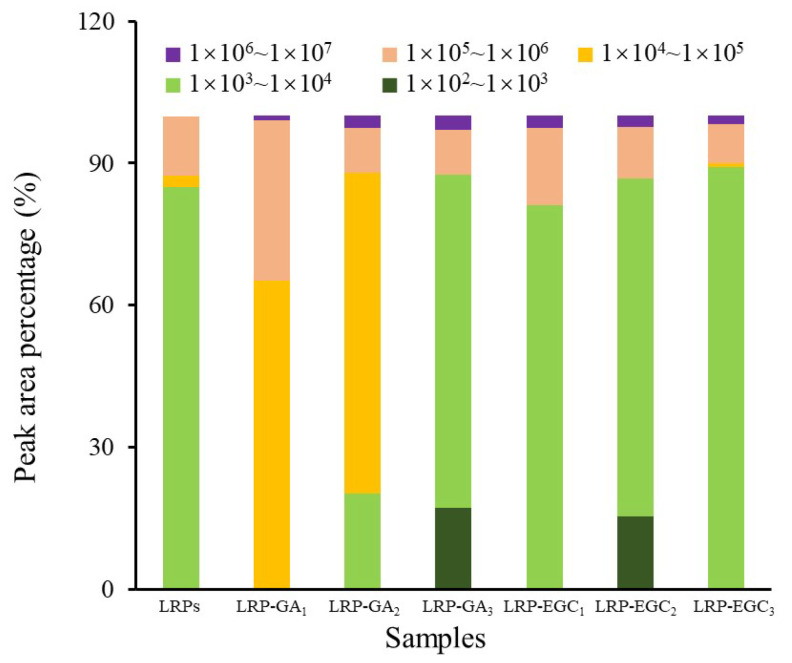
The peak area percentage of fractions with different molecular weights from LRPs and LRP-phenol complexes.

The elution profiles of LRPs and LRP-phenol complexes as a function of the elution time obtained by the HPSEC-MALL-RI method are presented in Figure 5, the retention time and peak area percentages of their fractions in different MW ranges are summarized in Table 2 and visually presented in Figure 6. The peak area percentage is proportional to the concentration of fractions, which can be used to calculate the weight-average MW. In general, the weight-average MWs of LRP-phenol complexes were significantly higher than those of LRPs. The peak area percentages of LRP fractions in the MW ranges of 1 × 10${}^{-3}$ × 10${}^{4}$, 1 × 10${}^{-4}$ × 10${}^{5}$, and 1 × 10${}^{-5}$ × 10${}^{6}$ were 84.9%, 2.5%, and 12.5%, respectively. Compared to LRPs, fractions in a higher MW range of 1 × 10${}^{-6}$ × 10${}^{7}$ were observed for LRP-GA complexes; fractions in the MW range of 1 × 10${}^{-4}$ × 10${}^{5}$ increased and in 1 × 10${}^{-3}$ × 10${}^{4}$ decreased for LRP-GA${}_{1}$ and LRP-GA${}_{2}$ complexes. Particularly, both had the lowest MW of 17.36 kDa and 6.79 kDa, respectively. This indicated that free GA was not found in LRP-GA${}_{1}$/GA${}_{2}$ complexes (the MW of GA is 306 Da). These results imply that GA was successfully combined with LRPs. The fraction of LRP-GA${}_{3}$ in the lower MW range of 1 × 10${}^{-2}$ × 10${}^{3}$ was observed, which could be due to the low binding ratio and aggregation of GA. LRP-EGC complexes also had fractions in the high MW range of 1 × 10${}^{-6}$ × 10${}^{7}$. By contrasting to LRPs, fractions in the ranges 1 × 10${}^{-5}$ × 10${}^{6}$ and 1 × 10${}^{-3}$ × 10${}^{4}$ increased for LRP-EGC${}_{1}$ and LRP-EGC${}_{3}$, respectively. Several studies [13,16] had similar results showing that polysaccharide-phenol complexes had different MW distributions with polysaccharides.

### 3.5. Antioxidant Activities of LRPs and LRPs-GA/EGC Complexes

**Figure 7:**
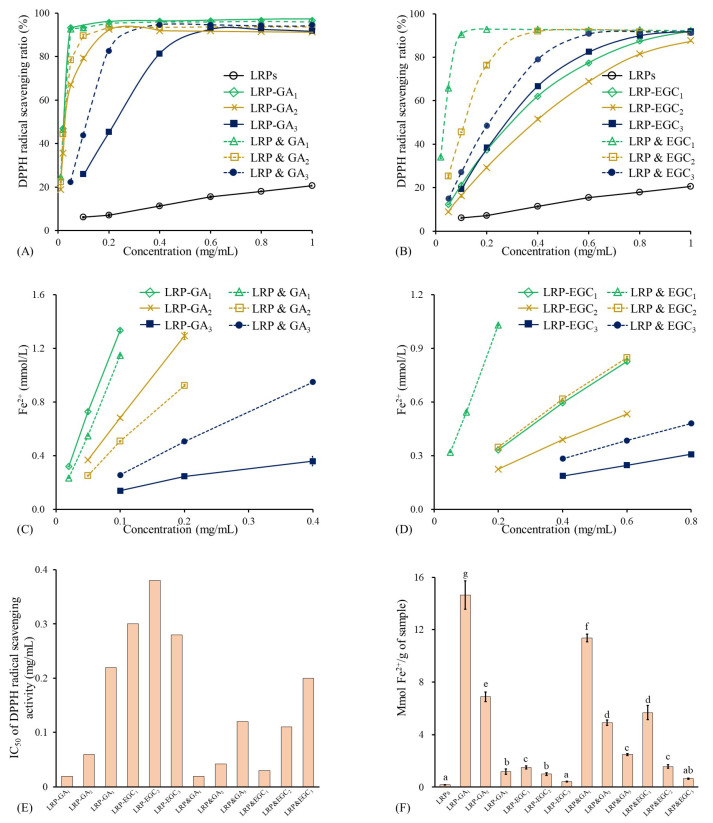
Antioxidant activities of LRPs, LRP-phenol complexes, and their physical mixtures. (**A**) DPPH radical scavenging ratio of LRPs, LRP-GA complexes, and their physical mixtures at different concentrations; (**B**) DPPH radical scavenging ratio of LRPs, LRP-EGC complexes, and their physical mixtures at different concentrations; (**C**) FRAP activities of LRPs, LRP-GA complexes, and their physical mixtures at different concentrations; (**D**) FRAP activities of LRPs, LRP-EGC complexes, and their physical mixtures at different concentrations. (**E**) IC${}_{50}$ values for DPPH radical scavenging activity of LRP-phenol complexes and their physical mixtures; (**F**) FRAP activities of LRPs, LRP-phenol complexes, and their physical mixtures expressed as mmol Fe${}^{2+}$/g sample. Means of the samples with different letters (a–g) differ significantly (*p* <0.05).

As shown in Figure 7A, the DPPH radical scavenging ratio of LRPs linearly increased with the increase in concentration and reached 20.6% at 1 mg/mL. The results are similar to those obtained by Su & Li. [38]. In general, LRP-GA/EGC complexes and their mixtures had significantly stronger DPPH radical scavenging activity than LRPs, which was due to the hydrogen-donating ability and redox property of GA/EGC (Figure 7A,B) [39]. Yong et al. [36] also reported that dialdehyde starch displayed enhanced antioxidant activity after grating with catechins. The IC${}_{50}$ values of LRP-GA${}_{1}$, LRP-GA${}_{2}$, LRP-GA${}_{3}$, LRPs & GA${}_{1}$, LRPs & GA${}_{2}$, and LRPs & GA${}_{3}$ were 0.02, 0.06, 0.22, 0.02, 0.04, and 0.12 mg/mL, respectively (Figure 7E). The higher binding ratio/mass ratio of GA in samples contributes to better DPPH radical scavenging activity. Moreover, LRP-GA${}_{2}$ and LRP-GA${}_{3}$ complexes showed less free-scavenging activity than their corresponding mixtures; this may be explained by the fact that the hydrogen bonds combine between LRPs with GA and consumption of hydroxyl groups. Similarly, LRP-EGC complexes exhibited lower DPPH radical scavenging ratios as well as higher IC${}_{50}$ values compared to their mixtures. Notably, there was a difference in DPPH radical scavenging activities between LRP-GA and LRP-EGC complexes, which was due to the different type and total content of phenol.

The antioxidant activities of LRPs, LRP-GA/EGC complexes, and their mixtures evaluated by FRAP are presented in Figure 7C,D,F. LRPs had the lowest FRAP value of 0.189 mmol Fe${}^{2+}$/g sample, showing their weak electron donation ability. Regarding LRP-GA/EGC complexes and their corresponding mixtures, the FRAP values increased with the molar ratio of GA/EGC increasing, suggesting that the phenolic compounds were mainly responsible for the electron donation even when complexed to the LRPs [40]. The FRAP values of LRP-GA${}_{3}$ and LRP-EGC complexes were lower than those of their corresponding mixtures, whereas LRP-GA${}_{1}$ and LRP-GA${}_{2}$ showed higher FRAP values than LRP&GA${}_{1}$ and LRP&GA${}_{2}$ mixtures, respectively.

### 3.6. Immunomodulatory Activity of LRPs-GA/EGC Complexes on RAW264.7 Cells

**Figure 8:**
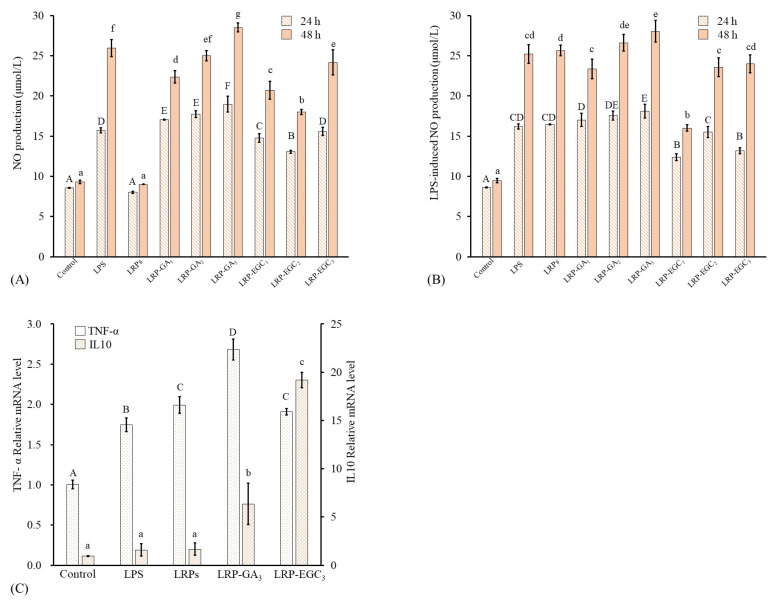
Macrophage-stimulating activities of LRPs and LRP-phenol complexes. (**A**) NO production of RAW264.7 cells stimulated by LRPs and LRP-phenol complexes; (**B**) NO production of LPS-induced RAW264.7 cells suppressed by LRPs and LRP-phenol complexes; (**C**) mRNA expression of cytokines (TNF-α, IL10) in RAW264.7 cells stimulated by LRPs, LRP-GA${}_{3}$, and LRP-EGC${}_{3}$. Means of the samples with different letters (A–F, a–g) differ significantly (*p* < 0.05).

NO is an important immune-regulatory signaling molecule whose release from macrophages can suppress the growth of bacterial infection or cancer cells [41]. Further, excessive production of NO can induce oxidative stress and inflammatory disease [42]. The levels of NO and LRP-induced NO production from RAW 264.7 cells treated with different samples for 24 h and 48 h are shown in Figure 8A,B. LRPs and LRP-GA/EGC complexes were used at same LRP concentration of 200 μg/mL. Cells treated with LPS at 500 ng/mL were applied as a positive group. Regarding cells incubated for 24 h, the level of NO produced from LRP-treated cells had no significant difference to that from the control; values were 8.58 and 8.01 μmol/L, respectively. The LRP-GA/EGC complexes and LPS all stimulated NO production as compared to the control, showing increasing NO production in the order: LRP-EGC${}_{2}$< LRP-EGC${}_{1}$< LRP-EGC${}_{3}$≤ LPS < LRP-GA${}_{1}$≤ LRP-GA${}_{2}$< LRP-GA${}_{3}$ at 13.05, 14.7, 15.59, 15.72, 17.0, 17.7, and 18.95 μmol/L, respectively. Combination with GA/EGC significantly increased the stimulation of LRPs, whereas the increase in the GA/EGC compound in complexes had no positive effect on NO levels, suggesting that LRPs in complexes play the major role in the stimulation mechanism. A similar phenomenon was observed for the cells incubated after 48 h. For cells stimulated by LPS, LRP-EGC complex showed an inhibitory effect on the LPS-induced NO production. Particularly, LRP-EGC${}_{1}$ and LRP-EGC${}_{3}$ significantly decreased the LPS-induced NO production for cells incubated after 24 h, and LRP-EGC${}_{1}$ showed a significantly inhibitory effect on LPS-induced cells after 48 h.

As mentioned above, LRP-GA${}_{3}$ and LRP-EGC${}_{3}$ showed relatively better immunostimulatory effects with NO production compared with other complexes. Here, the effects of LRPs, LRP-GA${}_{3}$/EGC${}_{3}$ complexes on mRNA expression of cytokines (TNF-α, IL10) in RAW264.7 cells were investigated. Compared with the control untreated cells, the mRNA expression of TNF-α increased by 1.65, 1.89, 2.54, and 1.81-fold when cells were treated with LPS, LRPs, LRP-GA${}_{3}$, and LRP-EGC${}_{3}$, respectively (Figure 8C), but the expression levels of anti-inflammatory cytokine IL-10 were elevated. Especially, LRP-GA${}_{3}$ and LRP-EGC${}_{3}$ increased IL-10 expression by 6.6- and 20.1-fold compared with the control. The IL-10 expression levels induced by LRP-GA${}_{3}$ and LRP-EGC${}_{3}$ were significantly higher than those stimulated by LRPs. The results show that binding with GA/EGC has great potential in improving the beneficial immunomodulation of LRPs on macrophages.

## 4. Conclusions

In this work, the binding interactions between GA/EGC and LRPs were studied by evaluating the effect of environmental factors (pH, temperature, and NaCl concentration) on the binding amount of GA/EGC to LRPs, and measuring the UV–Vis spectra, FTIR spectra, and MW of LRPs-GA/EGC complexes. It was demonstrated that GA/EGCs successfully bond to LRPs by hydrogen bond interactions, leading to the changes in structure and MW distribution of LRPs. The binding of phenolic compounds significantly increased the DPPH radical scavenging activity and FRAP capacity of LRPs, and the increase degree was dependent not only on the phenol type but also on the mass ratio of the phenols in complexes. Moreover, GA and EGC combined to LRPs promoted the NO production of macrophages in normal conditions; EGC with a mass ratio of 698.71 mg/g bound to LRPs showed inhibitory effects on the NO over-production stimulated by LPS. Binding with GA/EGC did not enhance the TNF-α production induced by LRPs, but LRP-GA/EGC (with binding ratios of 105.42 and 50.71 mg/g, respectively) significantly improved the expression of anti-inflammatory cytokine IL10 as compared with LRPs. These results contribute to knowledge on the interaction mechanism of LRPs and phenolic compounds, and provide evidence that binding with phenol is a promising way to improve the structure, antioxidant, and anti-inflammatory properties of LRPs. Further research into the bioaccessibility and stability of phenolic compounds bound to LRPs under different conditions (for example, gastrointestinal digestion, light, humidity, and so on) will be carried out to obtain more knowledge on the nutritional aspects of LRP-phenol complexes.

## Figures and Tables

**Table 1:** Preparation conditions of LRP-GA and LRP-EGC complexes with different binding amounts of GA/EGC: concentration of GA/EGC (mg/mL), mass ratio of LRPs to GA/EGC, pH, temperature.

Complex	Name	Preparation Conditions			
		Concentration of GA/EGC (mg/mL)	Mass Ratio of LRPs to GA/EGC	pH	Temperature (°C)
LRP-GA	LRP-GA${}_{1}$	8.0	1:4	7	0
	LRP-GA${}_{2}$	2.0	1:1	7	0
	LRP-GA${}_{3}$	0.5	4:1	7	0
LRP-EGC	LRP-EGC${}_{1}$	8.0	1:4	5	0
	LRP-EGC${}_{2}$	2.0	1:1	5	0
	LRP-EGC${}_{3}$	0.5	4:1	5	0

**Table 2:** The molecular weights of LRPs and LRP-phenol complexes detected by the HPSEC-MALL-RI method.

Sample	Retention Time (min)	Molecular Weight (Da)	Peak Area Percentage (%)	Average Molecular Weight (Da)
LRP	7.237–11.370	2.561 × 10${}^{5}$ (±1.031%)	12.5	3.618 × 10${}^{4}$
	11.370–12.244	8.771 × 10${}^{3}$ (±8.120%)	8.3	
	12.244–13.934	4.055 × 10${}^{3}$ (±10.646%)	26.8	
	13.934–16.950	2.782 × 10${}^{3}$ (±16.105%)	47.1	
	16.950–20.058	3.320 × 10${}^{4}$ (±29.285%)	2.5	
	21.204–23.165	8.040 × 10${}^{3}$ (±44.484%)	2.7	
LRP-GA${}_{1}$	7.156–8.877	1.540 × 10${}^{6}$ (±10.451%)	0.9	1.106 × 10${}^{5}$
	8.877–11.089	3.246 × 10${}^{5}$ (±40.231%)	5.8	
	11.089–12.283	1.736 × 10${}^{4}$ (±27.878%)	18.9	
	12.283–14.285	1.065 × 10${}^{5}$ (±39.474%)	28.1	
	14.285–17.411	2.357 × 10${}^{4}$ (±28.070%)	46.3	
LRP-GA${}_{2}$	6.842–8.870	1.350 × 10${}^{6}$ (±4.031%)	2.5	6.501 × 10${}^{4}$
	8.870–11.177	1.786 × 10${}^{5}$ (±19.428%)	9.6	
	11.177–12.296	6.790 × 10${}^{3}$ (±21.058%)	20.4	
	12.296–14.078	2.497 × 10${}^{4}$ (±31.497%)	25.3	
	14.078–16.980	1.505 × 10${}^{4}$ (±24.199%)	34.7	
	17.015–19.601	1.584 × 10${}^{4}$ (±11.543%)	7.5	
LRP-GA${}_{3}$	6.815–8.896	1.617 × 10${}^{6}$ (±1.334%)	2.9	6.663 × 10${}^{4}$
	8.926–11.038	1.749 × 10${}^{5}$ (±0.784%)	9.5	
	11.038–12.727	3.523 × 10${}^{3}$ (±11.649%)	35.5	
	12.727–14.024	1.291 × 10${}^{3}$ (±27.174%)	18.2	
	14.024–14.959	9.475 × 10${}^{3}$ (±17.222%)	17.3	
	14.990–17.493	8.874 × 10${}^{3}$ (±25.312%)	16.6	
LRP-EGC${}_{1}$	6.875–8.866	1.102 × 10${}^{6}$ (±1.355%)	2.5	4.691 × 10${}^{4}$
	8.866–11.279	1.016 × 10${}^{5}$ (±0.856%)	16.4	
	11.279–12.516	3.479 × 10${}^{3}$ (±15.877%)	37.9	
	12.516–13.964	3.221 × 10${}^{3}$ (±17.625%)	20.6	
	13.964–14.899	2.195 × 10${}^{3}$ (±12.611%)	13.7	
	14.899–16.317	4.688 × 10${}^{3}$ (±14.529%)	8.9	
LRP-EGC${}_{2}$	6.905–8.926	1.066 × 10${}^{6}$ (±1.419%)	2.4	7.895 × 10${}^{4}$
	8.926–11.128	1.213 × 10${}^{5}$ (±0.931%)	10.8	
	11.128–13.994	1.446 × 10${}^{3}$ (±17.875%)	62.7	
	13.994–14.959	9.786 × 10${}^{2}$ (±22.227%)	15.5	
	14.959–17.041	1.886 × 10${}^{3}$ (±31.802%)	8.6	
LRP-EGC${}_{3}$	7.026–8.806	2.169 × 10${}^{6}$ (±1.727%)	1.8	5.799 × 10${}^{4}$
	8.836–10.978	1.960 × 10${}^{5}$ (±2.232%)	8.3	
	10.978–13.964	3.035 × 10${}^{3}$ (±12.892%)	63.1	
	13.994–16.166	2.196 × 10${}^{3}$ (±16.236%)	26.1	
	16.166–17.403	2.424 × 10${}^{4}$ (±25.006%)	0.8	

## Data Availability

Data is contained within the article.

## Abbreviations

The following abbreviations are used in this manuscript:

GA	gallic acid
EGC	epigallocatechin
UV–Vis	ultraviolet–visible
FTIR	Fourier-transform infrared
MW	molecular weight
LPS	liposaccharide
DMEM	Gibco Dulbecco’s Modified Eagle Medium
PBS	phosphate-buffered saline
DPPH	2,2-diphenyl-1-picrylhydrazyl
DNS	3,5-dinitrosalicylic acid
HPSEC	high-performance size-exclusion chromatography
DPPH	2,2-diphenyl-1-picrylhydrazyl
FRAP	ferric-reducing antioxidant power
TNF-α	tumor necrosis factor-α
IL-10	interleukin-10
